# Characterization of the *STK11* splicing variant as a normal splicing isomer in a patient with Peutz–Jeghers syndrome harboring genomic deletion of the *STK11* gene

**DOI:** 10.1038/hgv.2016.2

**Published:** 2016-03-03

**Authors:** Kenta Masuda, Yusuke Kobayashi, Tokuhiro Kimura, Kiyoko Umene, Kumiko Misu, Hiroyuki Nomura, Akira Hirasawa, Kouji Banno, Kenjiro Kosaki, Daisuke Aoki, Kokichi Sugano

**Affiliations:** 1 Department of Obstetrics and Gynecology, Keio University School of Medicine, Tokyo, Japan; 2 Center for Medical Genetics, Keio University School of Medicine, Tokyo, Japan; 3 Department of Pathology, Yamaguchi University Graduate School of Medicine, Yamaguchi, Japan; 4 Oncogene Research Unit / Cancer Prevention Unit, Tochigi Cancer Center Research Institute, Tochigi, Japan

## Abstract

We report a *STK11* splicing variant comprising a 131-bp insertion that is derived from intron 1, which has previously been reported to possess potent pathogenicity. The same variant was detected in a Peutz–Jeghers syndrome patient harboring a genomic deletion in the vicinity of exon 1 of the *STK11* gene, which indicated that this variant was derived from the wild-type allele. We also found the same variant in other normal subjects. This variant corresponds to the predicted transcript variant of *STK11* (XM_011528209), which is derived from the genomic sequence of Chr19 (NT_011295.12). Therefore, we concluded that the splicing variant was not pathogenic.

Peutz–Jeghers syndrome (PJS) is an autosomal dominant disease characterized by intestinal polyposis, mucocutaneous pigmentation, and malignancies. Germline mutations in the *STK11 (LKB1)* gene have been identified as the major cause of PJS.^1,2^ *STK11* contains nine coding exons and one non-coding exon. STK11 is a 433 residue serine threonine protein kinase that controls the activity of AMP-activated protein kinase family members and has roles in cell metabolism, cell polarity, chromatin remodeling, cell cycle arrest, apoptosis, and DNA damage responses.^3^ Reportedly, half of the mutations in *STK11* have been identified as point mutations; however, a large genomic deletion has also been found in 30% of patients with PJS.^4^ Direct sequencing and multiplex ligation-dependent probe amplification are both recommended in the analysis of subjects with PJS. Splicing variants of *STK11* have also been suggested to be mutations that cause PJS.^5,6^

We herein report one *STK11* splicing variant found in a PJS patient, which was difficult to ascertain as a normal variant or a pathogenic form. The patient was a 30-year-old female who developed some hamartomas in the sigmoid colon, pigmentation on the oral mucosa, lips, and fingers, and cervical cancer, as we previously described.^7^ She had a genomic deletion comprising exon 1 in the *STK11* gene and had been diagnosed as PJS. Her pedigree is shown in Figure 1a. We also detected a *STK11* splicing variant in the reverse transcription-PCR (RT-PCR) products from the blood samples treated with puromycin. The variant contained a 131-bp insertion, which was derived from the middle part of intron 1 (9,064-bp downstream of exon 1), between exons 1 and 2 (Figure 2c). Without puromycin, no splicing variant was found in the patient by RT-PCR/direct sequencing (Figure 1b). Puromycin, a protein-translation inhibitor, is known to reduce the effects of nonsense-mediated mRNA (messenger RNA) decay (NMD); therefore, we expected the signals from this splicing variant to be suppressed by NMD.^8^ This splicing variant resulted in a premature termination codon in exon 2 (Figure 2d).

Because the pathogenic mutation in this patient comprised the genomic deletion of exon 1, as reported in Kobayashi *et al*., and the forward RT-PCR primer was located in this exon, no PCR product was amplified by RT-PCR. Therefore, this splicing variant was derived from the wild-type allele and assumed to be non-pathogenic (Figure 2d). This splicing isomer was not detected in RT-PCR using total RNA extracted from peripheral blood lymphocytes without the puromycin treatment.

Abed *et al*. have previously presented a case of PJS with a compound heterozygosity for *STK11* splice mutations. One mutation contained an A>G transition that inactivated the acceptor splice site consensus of intron 1 (*STK11* IVS1–2A>G). The other mutation was the same splicing mutation as in our case, which contained 131 bp derived from intron 1 that were inserted between exons 1 and 2. This mutation was shared and segregated with the patient’s son and daughter.^9^ Abed concluded that it was unclear whether this splicing form was deleterious or normal.

To address this question, we performed RT-PCR spanning exons 1–3 and direct sequencing for subjects who did not carry a germline mutation in *STK11*. In the RT-PCR analysis spanning exons 1 and 3, we found wild-type bands and weak upper bands by RT-PCR spanning exons 1–3 in four subjects (Figure 2a). The upper bands from the RT-PCR products were excised from the gel, and extracted DNAs were reamplified. The same splicing variant carrying the 131-bp insertion derived from intron 1 between exons 1 and 2 was detected in all subjects (Figure 2b). Because we also identified the same upper bands in other normal controls (data not shown), we concluded that the splicing variant was not pathogenic but was a normal splicing isomer.

In addition, immunohistochemistry using an anti-STK11 antibody recognizing amino acids 73–122 of human STK11 protein (ab58786; Abcam, Cambridge, UK) was performed in this patient to confirm whether the allele with this splicing variant expressed normal STK11 protein. The results showed that STK11 protein expression was not attenuated in the normal endometrium, but it was attenuated in cervical cancer tissue (Figure 1c). *STK11* is a tumor suppressor gene, which was originally assigned to the chromosomal locus showing a frequently deleted region of Chr19p13.3, implying that the inactivation of *STK11* occurs in the late stage of carcinogenesis.^10^ Signals from the splicing variant were difficult to detect by RT-PCR of the puromycin non-treated sample, implying the absence of the mutated protein. However, it may be possible to detect an aberrantly spliced protein harboring the peptide residues 73 through 97 of the STK11 protein, using this rabbit polyclonal antibody whose reactivity was thought to be weak (but present).

To establish the mechanism by which this splicing variant occurred, we analyzed the 131-bp sequence inserted in intron 1. Using the software RepeatMasker (Institute for Systems Biology, Seattle, WA, USA), long interspersed element-1 (LINE-1/L1) and Alu elements were identified in the 131-bp sequence.^11^ A previous study has reported that some L1 elements have multiple splice donor sites and splice acceptor sites, and lead to the aberrant splicing of genes,^12^ and we hypothesized that this splicing variant was caused by the GT–AG motif present in the L1 and Alu repeat in intron 1 of the *STK11* gene (Figure 2c). Another piece of evidence supporting our hypothesis that this phenomenon is caused by cryptic splice sites located in intron 1 and not allelic mutations has been submitted to a database as a putative transcript variant X2 (XM_011528209.1). This sequence, predicted by automated computational analysis, contains the same sequence as in our case, comprising 131 bp derived from intron 1 that was inserted between exons 1 and 2. This transcript variant X2 has its coding sequence located in the 131 bp derived from intron 1 and exon 2, and its mRNA is suppressed by NMD.

## Figures and Tables

**Figure 1:**
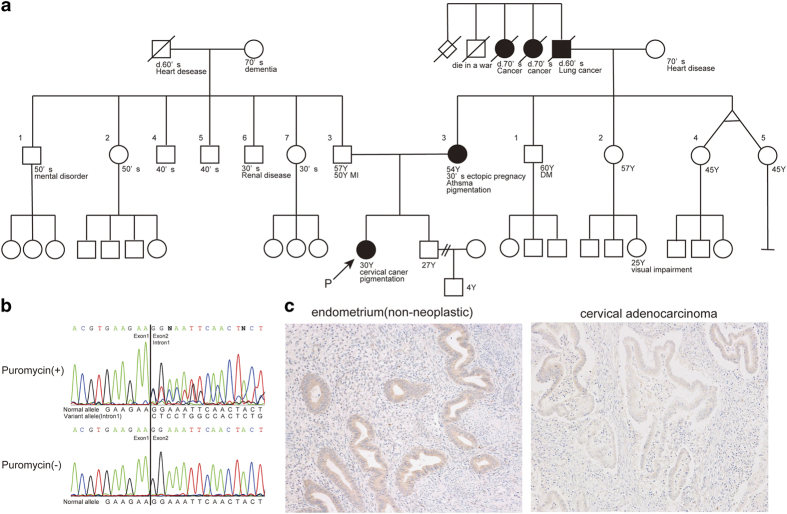
Genetic analysis of the *STK11* gene and protein expression in the Peutz–Jeghers syndrome (PJS) patient. (**a**) Pedigree of the PJS patient. (**b**) Electropherograms of the *STK11* splicing variant. The *STK11* splicing variant was identified in the PJS patient by reverse transcription-PCR/direct sequencing with puromycin (upper panel). No splicing variant was detected in the subject without puromycin treatment (lower panel). (**c**) Immunohistochemistry for STK11 in the normal endometrium (left) and cervical adenocarcinoma (right). STK11 protein localization was diminished in the cervical adenocarcinoma.

**Figure 2:**
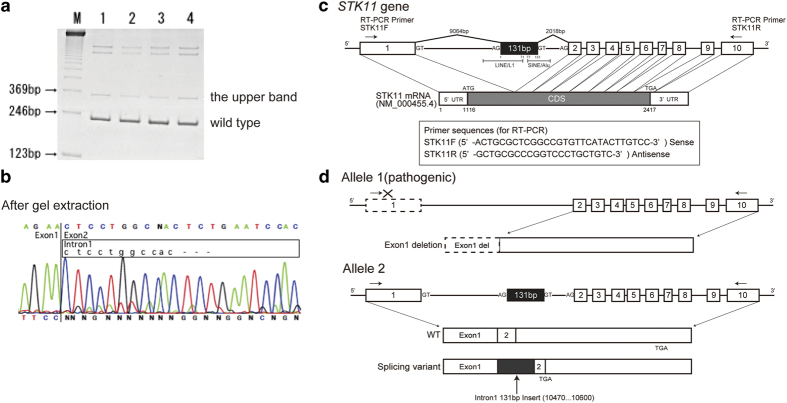
(**a**) Reverse transcription-PCR (RT-PCR) products encompassing *STK11* exons 1–3 were analyzed. All samples were pretreated with puromycin before RNA extraction. Each lane exhibits the wild-type band (236 bp) and upper band (367 bp). (**b**) Electropherograms of RT-PCR products after gel extraction of the upper band. The signal from the splicing variant was detected. (**c**) Genomic organization of the *STK11* gene and messenger RNA (mRNA) (NM_000455.4), whose coding sequence spans from 1,116 to 2,417 bp. The annealing positions and sequences of primers used for RT-PCR are shown. An AG-GT splicing motif sequence located in LINE/L1 and SINE/Alu elements worked as the cryptic splicing site, and the 131-bp sequence was spliced out as the aberrant RNA isomer in RT-PCR from puromycin-treated peripheral blood lymphocytes. The location of LINE/L1 or SINE/Alu element occupies 71 bp in the forward part or 55 bp in the backward part of the 131-bp fragment, respectively. (**d**) Patient’s *STK11* gene alleles. This patient had a genomic deletion of exon 1 in the *STK11* gene, as we previously described.^7^ Allele 1 shows an aberrant allele with an exon 1 deletion. Allele 2 shows a normal allele with the splicing variant that resulted in a premature terminal codon at exon 2. The annealing positions of primers used for RT-PCR are shown as arrows. The forward primer for RT-PCR cannot anneal to the exon 1-deleted mRNA.

## References

[bib1] Hemminki A , Markie D , Tomlinson I , Avizienyte E , Roth S , Loukola A et al. A serine/threonine kinase gene defective in Peutz-Jeghers syndrome. Nature 1998; 391: 184–187. 9428765 10.1038/34432

[bib2] Jenne DE , Reimann H , Nezu J , Friedel W , Loff S , Jeschke R et al. Peutz-Jeghers syndrome is caused by mutations in a novel serine threonine kinase. Nat Genet 1998; 18: 38–43. 9425897 10.1038/ng0198-38

[bib3] Yoo LI , Chung DC , Yuan J . LKB1--a master tumour suppressor of the small intestine and beyond. Nat Rev Cancer 2002; 2: 529–535. 12094239 10.1038/nrc843

[bib4] Aretz S , Stienen D , Uhlhaas S , Loff S , Back W , Pagenstecher C et al. High proportion of large genomic STK11 deletions in Peutz-Jeghers syndrome. Hum Mutat 2005; 26: 513–519. 16287113 10.1002/humu.20253

[bib5] Hastings ML , Resta N , Traum D , Stella A , Guanti G , Krainer AR . An LKB1 AT-AC intron mutation causes Peutz-Jeghers syndrome via splicing at noncanonical cryptic splice sites. Nat Struct Mol Biol 2005; 12: 54–59. 15608654 10.1038/nsmb873

[bib6] Launonen V . Mutations in the human LKB1/STK11 gene. Hum Mutat 2005; 26: 291–297. 16110486 10.1002/humu.20222

[bib7] Kobayashi Y , Masuda K , Kimura T , Nomura H , Hirasawa A , Banno K et al. A tumor of the uterine cervix with a complex histology in a Peutz—Jeghers syndrome patient with genomic deletion of the STK11 exon 1 region. Futur Oncol 2014; 10: 171–177. 10.2217/fon.13.180 24490603

[bib8] Nomura S , Sugano K , Kashiwabara H , Taniguchi T , Fukayama N , Fujita S et al. Enhanced detection of deleterious and other germline mutations of hMSH2 and hMLH1 in Japanese hereditary nonpolyposis colorectal cancer kindreds. Biochem Biophys Res Commun 2000; 271: 120–129. 10777691 10.1006/bbrc.2000.2547

[bib9] Abed AA , Günther K , Kraus C , Hohenberger W , Ballhausen WG . Mutation Screening at the RNA Level of the STK11/LKB1 Gene in Peutz-Jeghers Syndrome Reveals Complex Splicing Abnormalities and a Novel mRNA Isoform (STK11 c. 597^598insIVS4). Hum Mutat 2001; 18: 397–410. 11668633 10.1002/humu.1211

[bib10] Hemminki A , Tomlinson I , Markie D , Järvinen H , Sistonen P , Björkqvist AM et al. Localization of a susceptibility locus for Peutz-Jeghers syndrome to 19p using comparative genomic hybridization and targeted linkage analysis. Nat Genet 1997; 15: 87–90. 8988175 10.1038/ng0197-87

[bib11] Smit AFA , Hubley R , Green P. RepeatMasker Open-3.0. Available at http://www.repeatmasker.org.

[bib12] Belancio VP , Hedges DJ , Deininger P . LINE-1 RNA splicing and influences on mammalian gene expression. Nucleic Acids Res 2006; 34: 1512–1521. 16554555 10.1093/nar/gkl027 PMC1415225

